# Biomolecule-Functionalized Smart Polydiacetylene for Biomedical and Environmental Sensing

**DOI:** 10.3390/molecules23010107

**Published:** 2018-01-04

**Authors:** Eunae Cho, Seunho Jung

**Affiliations:** 1 Institute for Ubiquitous Information Technology and Applications (UBITA) & Center for Biotechnology Research in UBITA (CBRU), Konkuk University, 120 Neungdong-ro, Gwangjin-gu, Seoul 05029, Korea; echo@konkuk.ac.kr; 2 Department of Bioscience and Biotechnology, Microbial Carbohydrate Resource Bank (MBRC) & Center for Biotechnology Research in UBITA (CBRU), Konkuk University, 120 Neungdong-ro, Gwangjin-gu, Seoul 05029, Korea

**Keywords:** biomolecules, polydiacetylene, conjugation, sensor

## Abstract

Polydiacetylene (PDA) has attracted interest for use as a sensing platform in biomedical, environmental, and chemical engineering applications owing to its capacity for colorimetric and fluorescent transition in response to external stimuli. Many researchers have attempted to develop a tailor-made PDA sensor via conjugation of chemical or biological substances to PDA. Here, we review smart bio-conjugates of PDA with various biomolecules such as carbohydrates, lipids, nucleic acids, and proteins. In addition, materialization and signal amplification strategies to improve handling and sensitivity are described.

## 1. Introduction

**Figure 1:**
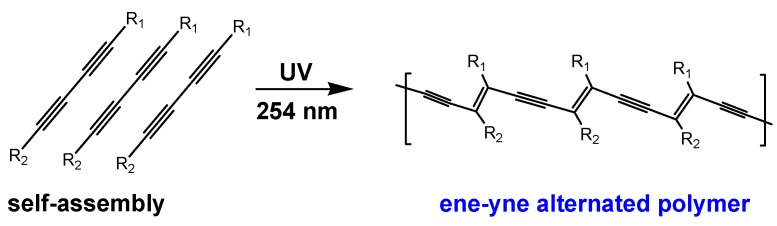
Polydiacetylene (PDA) synthesis scheme via self-assembly and polymerization of diacetylene monomers.

Polydiacetylene (PDA), first prepared by Wegner in 1969, can be easily synthesized by polymerization of diacetylenic monomers under photo-irradiation, free radical induction, or plasma treatment [1,2,3]. In general, when adjacent diacetylenes are stacked at a distance of about 5 Å and the angle of monomer with stacking axis is about 45°, 1,4-addition-polymerization is possible by UV light (254 nm) at room temperature without the need for a catalyst or initiator, and thus, no by-products are produced (Figure 1) [4]. The resultant PDA has an ene-yne alternating backbone structure and a deep blue color (absorption uv_max_ = ~640 nm), and the extended π-electron delocalization gives it excellent electronic and optical properties. When the blue-phase PDA encounters heat, organic solvent, mechanical stress, or molecular recognition, a blue-to-red color shift (the wavelength shift of maximum absorption from *ca.* 640 to *ca.* 540 nm) occurs and fluorescence switches on [5]. The switch is induced by a decrease in the effective conjugation length of the PDA backbone under lipid chain distortion and disorder. Based on these core characteristics, many scientists have attempted to apply PDA assembly to various biomedical, environmental, and nanotechnological applications [6].

For structural variation, the length of the acyl chain or the diacetylic position can be changed. Although 10,12-pentacosadiynoic acid (PCDA), 10,12-tricosadiynoic acid (TRCDA), 5,7-docosadiynoic acid (DCDA), and 5,7-tetracosadiynoic acid (TCDA) are all utilized as monomeric units [1], PCDA is the most widely used monomer. Given this basic unit, head group modification has been used to endow the polymer backbone with specific functions. In this respect, intelligent design using the PDA platform is achievable and valuable for researchers in many disciplines. Although various groups and methods for tethering to the diacetylene lipid have been investigated, this review focuses on the combination of biomolecules and PDA. These hybrid materials possess bio-induced specificity combined with the robustness/stimuli-responsiveness of a synthetic polymer.

**Figure 2:**
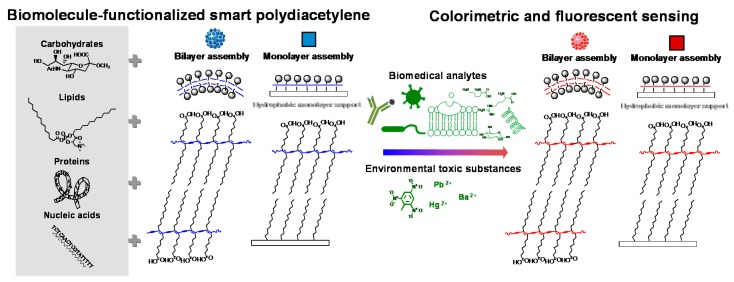
Biomolecule-functionalized polydiacetylene (PDA) based biomedical and environmental sensing.

Biomolecules include carbohydrates, lipids, proteins, nucleic acids, and small molecules such as vitamins, hormones, and metabolites. They are associated with various molecular interactions under physiological conditions, and their interactions macroscopically impact our body. For example, hemagglutinin anchored in the influenza viral envelope specifically binds the sialic acid terminating glycan on the surface of cellular membranes in a human host, permitting viruses to replicate inside the body, causing infection [7]. Similarly, bacterial toxins interact with a ganglioside moiety on host cells, eventually giving rise to inflammation and other related diseases via invasion [8,9]. In addition, biomembranes, which are comprised of lipids, carbohydrates, and proteins, have a dynamic structure, participating in numerous interfacial and cellular processes [10]. Focused on these biological issues, PDA has been modified and applied in various sensing fields (Figure 2). Biomolecule-functionalized PDA shows promise for many biomedical and environmental applications. Herein, this review deals mainly with the PDA bio-conjugates producing sensing signal, while the physicochemical aspects of PDA materials are not critically covered in this review.

## 2. Carbohydrate-Functionalized Polydiacetylene (PDA)

The first PDA sensing system using the viral lectin and hemagglutinin-sialic acid interaction was developed by Charych et al. [11]. Sialic acid, a neuraminic acid derivative with a nine-carbon backbone, is ubiquitous in animal cells. This sensor provides a direct colorimetric method for detection of influenza using a 2–5% sialoside PDA monolayer coated on an octadecylsilane layer. The sialoside group is attached to a diacetylene monomer with a triethylene glycol linker, and the sialoyl lipid is utilized for the liposome and Langmuir–Blodgett (LB) film–type PDA influenza virus sensor [11,12,13]. Sensing specificity has been demonstrated using a competitive inhibition assay with lactose-attached lipids and bovine serum albumin [11]. The mechanism of the thermochromic blue-to-red shift of the modified PDA thin film was also investigated at the molecular level [14].

Mannose-derivatized PCDA has been utilized to create a PDA LB film that can recognize *Escherichia coli* [15]. Even in the incorporation of mannoside lipid (MC_16_) into PDA, the color change was triggered by the specific bio-interaction of mannose and a toxin secreted by *E. coli* [16]. Interestingly, the color response could be controlled and monitored in-situ by a third factor (TiO_2_ sterilization colloid). While the mannose- and lactose-tethered diacetylenic monomers, through click reaction, were synthesized with different arms, only the mannose-linked PDA displayed a color transition in the presence of concavalin A. Furthermore, the longer spacer was more effective for the colorimetric response, permitting the optimal disposition of mannose for interaction with the lectin, and a longer arm acting as a lever causes more stress on the PDA backbone [17].

Recently, oligosaccharides have been incorporated into PDA assembly and applied to the detection of small molecules [18,19,20]. In 2015, succinoglycan monomer 1, isolated from *Sinorhizobium meliloti,* was directly derivatized to PCDA. The resulting modified PDA liposome exhibits color change and fluorescence within 1 min in the presence of some highly hydrophobic flavonoids [18]. In this case, the succinoglycan octasaccharide functions as a flexible molecular agent for capture of alpha- and beta-naphthoflavones. Subsequently, succinoglycan octasaccharide-functionalized PDA-doped alginate beads were developed for sensing ions of the toxic heavy metal, barium [19]. Since the succinoglycan octasaccharide is a pyruvyl and succinyl substituted linear glycan, the carboxyl and hydroxyl groups contribute to multilateral interactions of succinoglycan octasaccharide with Ba^2+^, producing a color signal via the triggering of the PDA array. For the cyclic oligosaccharide, a β-cyclodextrin-functionalized PDA vesicle was prepared, allowing selective visualization of cationic amino acids (arginine and lysine) among twenty amino acids [20]. Although the detection limit is not low (millimolar concentration), the cavity type β-cyclodextrin working as a membrane channel for target molecules is noted.

Some researchers have attempted to improve the time-consuming synthesis of carbohydrate-attached diacetylene lipids. If the interacting glycolipid itself is incorporated into the diacetylenyl assembly, interacting analytes can be detected. For example, G_M1_ and G_T1b_ gangliosides, which are present on the surface of intestinal cells and at neuromuscular junctions, were utilized for color-based detection of cholera toxin and botulinum neurotoxin [21,22]. For this application, 5% ganglioside lipid was used, since a higher concentration inhibits polymerization of the diacetylene assembly. This modified PDA system mimics cell membranes, and could be used to detect toxin translocation. Next, the inexpensive dioctadecyl glycerylether-β-glucoside was co-assembled into PDA, and *E. coli* detection was achieved [23]. *E. coli* was also recognized with 70–90 colorimetic response (CR%) within 20 s, using a PDA platform consisting of 2,4-heneicosadiynoic acid (HCDA) and a cholesterol-linked glucoside [24]. This report also notes the influence of spacer length, in which a longer spacer is more sensitive in detecting *E. coli*. In another study, the size effect was investigated in concanavalin A detection using *N*-acetamide-β-glucoside; smaller PDA vesicles showed stronger detection ability [25]. This effect is explained by steric hindrance and a higher rate of membrane rupture derived from the greater curvature of small PDA vesicles. The *E. coli* and glycolipid binding event was also successfully transmuted into color change using a combined liposome comprised of phospholipids:PCDA:glycolipid (1,2-dihexadecanoyl-3-*O*-β-maltotriosyl-glycerol) 5:5:0.2 [26]. In another example, multi-head glycolipids analogous to the native cell surface were embedded in the PDA array, where a complex PDA system of 5% G_M1_/5% sialic acid derivatized PCDA/90% PCDA was reported to target choleratoxin [27]. In a similar way, this combination surface system gave rise to good selectivity, with a 10 ng/mL detection limit for avian influenza, owing to the synergetic effect of the active sialic acid-β-glucoside and the inactive lactose-β-glucoside glycolipids [28]. In a PDA system, size and charge of head-groups can affect the color of the polymer due to the differential packing properties, planarity, and conformation of PDA [1,29,30]. Considering the sensor application, blue-phase PDA system is desirable for the starting material [31]. Thus, it is also important to use the appropriate portion of biomolecules for the construction of PDA bio-conjugate as a sensing platform. The carbohydrate-functionalized PDAs are listed in Table 1.

## 3. Lipid-Functionalized PDA

Other lipid moieties can be incorporated into PDA assembly, and lipid decoration is generally performed without covalent attachment (Table 2). Overall, the lipid portion inserted in the PDA system is larger than the carbohydrate portion, since the lipids interact well with diacetylene lipids. Interestingly, 40% dimyristoyl phosphatidylcholine (DMPC)/PDA vesicles display a color response in the presence of phospholipases [32,33]. The insertion of sphingomyelin and cerebroside into PDA also permits detection of sphingomyelinase and galactosidase [34]. These interfacial catalysts are ubiquitous in biomembranes, and the prepared systems can be used to detect the lipolytic process with the naked eye. The phospholipase action was detected on the silica microbead without nonspecific color change using PDA/DMPC and CaCl_2_ [35]. Enzyme inhibitor assay using this system also provides potential for novel therapeutic insight.

Furthermore, various combinations of phospholipids (DMPC, dimyristoyl phosphoethanolamine (DMPE), dimyristoyl phophatidylglycerol (DMPG), and cardiolipin) and PDA have been utilized for the detection of antimicrobial peptides (mellitin, maganin, and alamethicin) [36,37]. The peptide-membrane association has a direct relationship with the blue-to-red transition. In phosphatidylinositol-4,5-phospholipids (PIP_2_)/PDA systems, aminoglycosidic antibiotics have been visually and fluorescently expressed with a detection limit of 61 ppb for neomycin [38]. The mechanism follows the inhibition of PIP_2_-phospholipase C signaling pathway by PIP_2_ binding of neomycin. This biomimetic membrane system was also used to study penetration enhancers, membrane binding of casein oligomers, α-lactoalbumin, and β-lactoglobulin [39,40,41]. The effect of the physiological lipid molecule’s cholesterol and cardiolipin on membrane fluidity and thermal stability has been investigated using this biomimetic lipid/PDA system [42]. Using cholesterol-incorporated PDA, the bacterial pore-forming toxin streptolysin O was colorimetrically detected [43]. Since Streptolysin O (SLO) creates pores in biomembranes in a cholesterol-dependent manner, PDA surface disruption can be visually detected in a cholesterol-containing biomimetic PDA composition. Jelinek et al. have also evaluated the extent of membrane interactions of lipoproteins using a lipid/PDA platform [44]. In cases of disease related to lipid oxidation, reduced membrane–lipoprotein interactions may be a novel marker for oxidative stress-related diseases or provide a therapeutic methodology.

Glass-supported lipid (DMPC, sphingomyelin, cholesterol)/PDA films have been employed for visual detection of membrane active molecules such as polymixin B and for bacterial fingerprinting [45,46]. Further, to diagnose human diseases, an array-based lipid/PDA gel was developed, and the blue-to-red transition was induced by plasma molecular components [47]. An omics-based approach can clearly distinguish disease states using a prepared robust and stable sensing gel matrix. For materialized PDA systems, the color change can be determined using a digital color analysis (DCA) algorithm instead of CR%.

Conventional PDA solutions employ nano-sized vesicles, but micrometer-sized giant phospholipid/PDA vesicles have also been utilized to detect and analyze membrane processes [48]. This system does not require sonication of the lipid suspension in water and provides easy analysis using optical and microscopic methods. The time course of vaccinia virus internalization was traced using fluorescence microscopy imaging. Local interactions of vesicles with polymyxin B or uniform interactions of the membrane with lipophilic pharmaceuticals could also be observed. Used not only for a specific purpose, membrane lipid components are also generally co-incoporated into various PDA platforms to mimic biomembrane condition or modulate the PDA assembly.

## 4. Protein (Antibody) Functionalized PDA

Antibodies protect cells against foreign antigen by recognizing antigens with high affinity and selectivity [49]. Specific molecular recognition is achieved by noncovalent interactions, including hydrophobic interactions, Van der Waals forces, electrostatic interactions, and hydrogen bonds. The interaction can be visualized by using hybridization of antibodies with a PDA domain. Jiang et al. reported a chromatic immunoassay based on antibody-functionalized PDA [50]. Vesicles of DMPC/TRCDA were linked with goat anti-h-IgG via 1-ethyl-3-(3-dimethylaminopropyl)carbodiimide/*N*-hydroxysuccinimide (EDC/NHS) coupling, bovine serum albumin (BSA) blocking was used to block non-specific absorption, and then polymerization was performed. The resulting PDA produces a discernible color change in the presence of 1 ng/mL antigen. Recently, influenza detection was conducted using antibody-modified PDA [51,52]. The antibody for the H5 influenza virus was modified using EDC/NHS coupling after polymerization of diacetylene vesicles [51]. In another case, the antibody was conjugated with NHS-functionalized PCDA [52]. The modified vesicle and polydiacetylene difluoride (PVDF) supported one were polymerized. Although antibody conjugation methods differ slightly by case, DMPC was incorporated for enhanced sensitivity.

Signal improvement could also be obtained by using an ethylenediamine interlinker between individual antibody-PDA liposomes for target pathogens [53]. Subsequently, a hybrid stimulus strategy was introduced by the same group (Sim et al.), in which the primary response by a monoclonal antibody-conjugated PDA chip was amplified by the force generated by polyclonal antibody-conjugated magnetic beads, for prostate cancer detection [54]. The resulting sensitivity was as low as 0.1 ng/mL of the target complex. Employing a similar principal, polyclonal antibody–conjugated gold nanoparticles were utilized for signal amplification in micro-arrayed PDA sensors for human immunoglobulin E (hIgE) detection, amplifying the signal up to 100-fold over the primary response [55]. The mechanical stress was provided by the magnetic force.

To detect phophinothricin acetyltransferase (PAT) in genetically modified crops, a microbead-assisted PDA sensor tethering anti-PAT was developed [56]. DMPC/TRCDA vesicles were immobilized on amine-coated silica beads, coated with anti-PAT using EDC coupling, and protected using ethanolamine, followed by polymerization. With respect to the same antibody, a recent study suggested another immunohydrogel bead–type PDA sensor using a polyethylene glycol diacrylate hydrogel matrix [57]. Anti-hepatotoxin microcystin-leucine-arginine (MC-LR) was also conjugated to PDA using EDC/NHS, and the PDA immunosensor showed a detection limit of 1 ng/mL, which is the maximum content recommended by the World Health Organization (WHO) for human drinking water [58]. *Salmonella* detection was performed using EDC crosslinked antibody-PDA vesicles [59]. Several kinds of immunoglobulin-PDA-phospholipids, synthesized using another cross linker sulfosuccinimidyl 4-(*N*-maleimidomethyl)cyclohexane-1-carboxylate (sulfo-SMCC), were encapsulated in hydrophilic silioxane sol-gels for use as a solid-state colorimetric biosensor [60].

**Figure 3:**
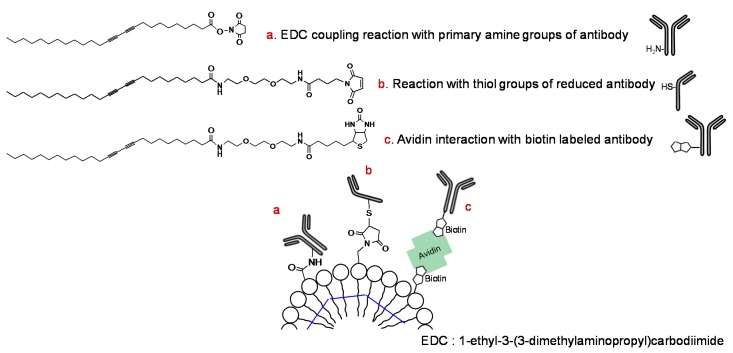
Representative derivatization of 10,12-pentacosadiynoic acid (PCDA) for antibody conjugation.

Although conjugation generally employs the amine group of the antibody protein, a few reports have described methods using the sulfhydryl group of the antibody and PCDA-maleimide (Figure 3) [61,62]. The prepared PDA vesicle was coated on nanoporous membranes such as PVDF, mixed cellulose esters (MCE), cellulose nitrate (CN), nylon, and polycarbonate (PC) [62]. Coating was conducted using a syringe and positive pressure with possible pretreatment of polylysine. Furthermore, a noncovalently linked antibody-PDA array system was developed using the avidin-biotin interaction (Figure 3) [63].

## 5. Protein (Peptide or Amino Acid)-Functionalized PDA

**Figure 4:**
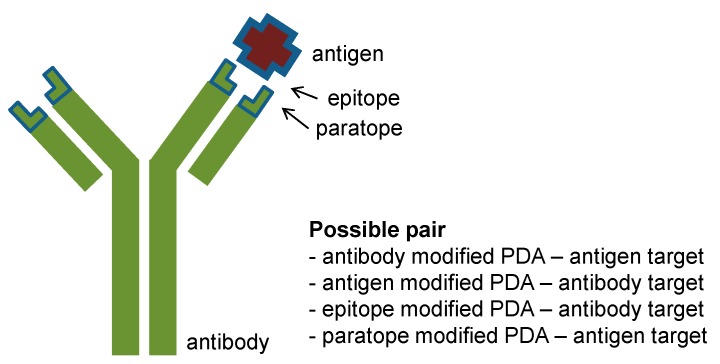
A schematic representation of antibody and antigen for conjugation with PDA.

The reverse type of PDA-based immunosensors can also be designed, using antigen- (or epitope)-conjugated PDA for detection of antibodies [64]. For example, an epitope-displaying peptide (c-myc-L_7_A_7_K_4_G) was synthesized, with the epitope displayed at the *N*-terminus of a hydrophobic and helical peptide. The epitope/DMPC/PDA system showed a color change in the presence of an antibody (0–100 μg/mL), and this platform provided rapid colorimetric detection compared with a conventional ELISA assay. In 2012, instead of an epitope-displaying peptide, a sensor was produced using an antigen (bovine viral diarrhea virus, BVDV, ~53 kDa) modified at the PCDA-epoxy with a linker [65]. The PCDA-epoxy/dimyristoyl-l-α-phosphatidic acid (DMPA)/BVDV antigen system exhibited better sensitivity (0 ng–100 μg/L) to BVDV IgG antibody (~150 kDa), and selectivity was confirmed using another antibody (100 μg/L). This system employs the small ligand-large target receptor concept, which generates a greater stress on the PDA backbone. There is still room for improvement in terms of direct detection, colorimetic response (CR%), and the detection time before it is ready for application in a real sensing format. Kim et al. expanded the idea in their next report using an influenza A virus M1 peptide tethered PDA liposome microarray [66]. The resulting detection limit was 2^−2^ Hemagglutinating Units (HAU) based on red fluorescence emission. They suggested that the steric repulsion between probe and target is more important for PDA sensing signal amplification than binding strength. This viewpoint is similar to the sialic acid functionalized PDA system for influenza hemagluttinin detection [11]. Sialic acid–mimic peptides have been developed for antiviral therapy via multiple serial selection using phage display technology [67]. The resulting pentadecapeptide (Fmoc-ARLSPTMVHPNGAQP-NH_2_) for pandemic H1N1 virus was conjugated to *N*-hydroxysuccinimide (NHS)-PCDA:PCDA at a 1:9 ratio [68]. This strategy was advanced in the above-mentioned antibody, antigen, and epitope modified PDA systems (Figure 4). In addition, paratope-functionalized PDA also shows promise as a novel sensing platform.

Other proteins, such as hexokinase and ionophores, have been incorporated into PDA assemblies to detect glucose and specific cations, respectively [69,70]. The first example to utilize protein conformational change for a solid-type PDA sensor was published in 1997 [69]. Hexokinase (M_w_ ~51,000) functioning as an induced fit enzyme was conjugated to 1:1 PCDA:NHS-PCDA, and polymerized for detection of glucose. For ionophore-modified PDA, valinomycin and monesin were added to PDA composed of DMPC:TRCDA 4:6, permitting detection of the physiologically important K^+^/Na^+^ [70]. The blue-to-red color transition is highly dependent on the cation-ionophore pair which is related in the microviscosity change in the PDA matrix. The cations or glucose were detected in the range of millimolar concentration, and the high concentration range is regarded to be due to the target and probe size effects mentioned above. Alternatively, it is thought the conformational change before and after target binding might not be enough to perturbe the PDA backbone. Unstructured proteins undergoing induced conformational change on binding can be effective for coupling with the signaling domain, PDA.

Small peptides have also been used for the construction of PDA-based sensor systems. A specific tripeptide tryptophan-histidine-tryptophan (WHW) was funtionalized to PDA, permitting detection of trinitrotoluene (TNT) explosives via multivalent binding mode [71]. Although the specific sequence for the TNT recognition motif was known to be WHWQRPLMPVSI based on phage display [72], only the truncated tripeptide PDA showed a colorimetric response. When using the full sequence, the large distance between the recognition site and the PDA backbone might pose an obstacle for electronic band change in the PDA backbone. The major driving force was considered to be pi-pi interactions between tryptophan and TNT’s aromatic ring. For sensitive and selective on-time monitoring, the system was further fused with single-walled carbon nanotube field-effect transistors (SWNT-FET) [73]. The combinational platform reaches 1 fM sensitivity. In addition, a mixed PDA assembly conjugated with fluorescent pentalysine and histidine was designed to mimic antibacterial (polymixin B)-lipopolysaccharide (LPS) interactions [74]. This system was utilized as a turn-on fluorescent sensor to detect LPS, a unique glycolipid produced by gram-negative bacteria, at low micromolar concentration. In a very recent study, a lysine- and arginine-rich pentapeptide, arginine-lysine-alanine-arginine-lysine (RKARK) was conjugated to TRCDA, producing two hydrophobic diacetylene chains at the *C*-terminal glutamic acids [75]. Based on its cell-penetrating properties, the cationic peptide-diacetylene permitted cell imaging by the polymerization that occurred during incubation with HeLa cells. In particular, endosomal uptake or membrane imaging could be controlled by different morphologies formed depending on the concentration; the assembly shows good biocompatibility.

Specific amino acid–derivatized PDA function in identification of LPS produced by different bacteria [76]. The amino acids tryptophan and tyrosine, which are essential residues for carbohydrate-binding proteins, were selected [77]. Glycine-linked PDA also exhibits a colorimetric response to Pb^2+^, and when embedded in a polyacrylonitrile nanofibrous membrane, it can be applied as a colorimetric strip [78]. Recently, histidine-functionalized PDA was reported, optimized for pH-responsive small interfering RNA (siRNA) delivery [79]. When conjugated to PDA with a trioxatridecane linker, the designed structure displayed a distinctive function compared with primary amine–linked PDA.

## 6. Nucleic Acid Functionalized PDA

Single-stranded deoxyribonucleic acid (DNA) and ribonucleic acid (RNA) hybridize with complementary DNA or RNA. When a specific probe sequence is given (DMPC:TRCDA:probe sequence-cholesterol 29:70:1), a target sequence can be detected via hybridization [80]. Although the nucleic acid is not directly linked to TRCDA, 20 nM target DNA was detectable using the naked eye. As a practical application of specific DNA-RNA hybridization, a sequence-specific DNA probe was conjugated to the surface of a PDA vesicle on an α-cyclodextrin-coated chip [81]. After polymerization, bacterial cells with the target 16S ribosomal RNA (rRNA) were detected on this PDA biochip, which provides a reliable and label-free pathogen detection method.

Furthermore, given that a binding event can create a new structure, guanine-rich single-stranded DNA (ssDNA) is of interest because it forms a quadruplex upon binding with K^+^ [82]. Kim et al. have developed solution- and solid-type G-rich ssDNA-modified PDA via direct conjugation, which produces anionic repulsion at the PDA surface upon binding with K^+^ [83]. The platform exhibits selective potassium ion detection in the range of sub-millimolar concentrations without interfering with sodium ions (the physiological concentration of potassium ions is 3.5–5.3 mM). Following that, selective and sensitive Hg^2+^ detection was achieved using a PDA microarray conjugated with a thymine-rich ssDNA aptamer (5′-TTCTTTCTTCCCCTTGTTTGTT-3′) [84]. The detection limit was 5 μM, which is much lower than the previous potassium detection range. An aptamer-based strategy was advanced using a bidentate aptamer-modified PDA for detection of thrombin protein [85]. The enhanced sensitivity and specificity was achieved by multisite binding and aggregation at a micromolar concentration. Another PDA-based aptasensor was designed targeting LPS of *E. coli* O157:H7 [86]. Using this aptamer-based colorimetric sensor, a range of 10^4^–10^8^ colony-forming units (CFU)/mL was specifically detected. Recently, Zn(II)-specific aptamers were also conjugated to PDA, to develop a PVDF strip aptasensor [87]. From the results, it is suggested that the increased aptamer length and unfolding of the hairpin aptamer cause greater steric repulsion to increase sensor sensitivity.

## 7. Other Biomolecule Functionalized PDAs

Other small molecules are useful to functionalize PDA for specific interactions and detection systems. Biotin, soluble vitamin B_7_, binds to tetrameric avidin with high affinity (K_a_ = 10^15^ M^−1^) [88]. A biotin-modified PCDA with an ethylene oxide spacer was designed, and streptoavidin interaction was colorimetrically detected with a large insoluble cluster by tetrameric association [89]. Moreover, this special interaction has been utilized for immobilization of PDA vesicles. For example, streptoavidin-functionalized micropatterned PDA chips have been fabricated using biotinyl PCDA liposomes immobilized on a microchip [90]. Biotin-labeled target DNA transmitted the biotin-avidin interaction into fluorescence for pathogen detection. In this case, since a specific target sequence is not utilized, it can be universally applied to nucleic acid amplification–related detection systems in the fields of infectious diseases, forensic medicine, or genetically modified organisms. Another vitamin, B_9_ or folate, was conjugated to PDA liposomes for tumor imaging and targeted drug delivery [91]. Since the surface of cancerous cells over-express folate receptors, folic acid is a good ligand for targeted drug delivery [92]. In this respect, folic acid was modified with dodecylamine, and it forms folate-PDA liposomes (egg phosphatidylcholine:PCDA:folate dodecyl amine 8:1:0.8). This novel system supports future theragnosis showing high docetaxel encapsulation as well as cancer cell-targeted fluorescence imaging. Biotinylated PDA might be an alternative to folated PDA, considering that biotin is a growth promotor.

The neurotransmitter dopamine has been used as a simple PDA sensor for lead (II) ions [93]. Based on the lead catecholate complex, co-assembly of dopamine-linked PCDA and PCDA permitted colorimetric and fluorogenic detection of Pb^2+^. This PDA liposome was further immobilized on alginate hydrogel microbeads. The hydrogel support added stability and sensitivity to the solution-based PDA platform, and the resulting detection limit was 200 ppb for lead ions. For detection of another toxic heavy metal, Hg^2+^, the nucleotide base thymine was incorporated into a PDA array [94]. T-Hg^2+^-T bonding creates liposomal aggregation on a 0.45-μm filter film, which permits naked-eye detection with a sensitivity of 0.1 μM. This system does not display any color change, but shows blue aggregated spot in the presence of 0–10 μM of Hg^2+^. Taken together, small targeting moieties such as arginine-glycine-aspartic acid RGD, partial aptamers, and hormones have the potential for use in design of PDA-based sensors.

## 8. Conclusions and Outlook

This review summarized the progress in biomolecular functionalization of PDA. In terms of biomimetics, membrane interfacial events have been successfully realized by combining PDA with biomolecules. This improves our understanding of the organization of complex biological components and cellular processes. Above all, the shift from the invisible nano-world to the visible range remains attractive, and worthy of further exploration. This study guides us in developing new sensor platforms for biomedical and environmental applications. Since the stimuli-responsive color or fluorescence change property of PDA are successfully linked to bio-sourced macromolecules and small molecules, bioanalytical and biocompatible sensing tools can be fabricated. Identification of good combinations related to various cellular interactions and reactions will permit consistent development of novel sensor systems. In addition, the approach used in protein-PDA conjugates can be attempted in carbohydrate-PDA or lipid-PDA systems. To date, macromolecular proteins and nucleic acids have been utilized to modify PDA assemblies, due to their specific interactions with target molecules. Thus, the use of macromolecular carbohydrates such as polysaccharides may open a new area of PDA work. Although carbohydrates are crucially associated with various cellular interactions and recognition complexes, the potential, except for monosaccharides, has been overlooked. In addition, fusion with phage display and systematic evolution of ligands by exponential enrichment (SELEX) technology will permit fabrication of specific peptide and nucleic acid sequences for effective conjugation to PDA. Accordingly, technological support for carbohydrate sequence generation may make up for the gap with other biomolecule-conjugated polymers containing PDA. Another pioneering work of biomolecule-PDA conjugation is development as a theragnostic tool. PDA vesicles have an interior, which can encapsulate other drugs or active ingredients, as well as a fluorogenic exterior. To date, the functionality of the membrane itself has been the focus, and the inner portion shows promise for co-use for future work on PDA in theragnosis and monitoring. Without doubt, smart PDA materialization will also enrich studies on fused synthetic polymers-biopolymers.

## Figures and Tables

**Table 1:** Components for carbohydrate functionalized PDA system.

Active Component	Analytes	Chemical Structures	Ref
Sialic acid	virus	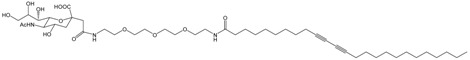	[11,12,13,14,21,27]
Mannose	*E. coli*	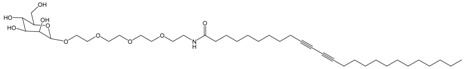	[15]
	*E. coli*	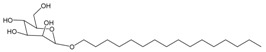	[16]
	lectin	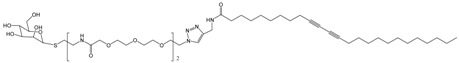	[17]
Succinoglycan octasaccharide	flavonoids	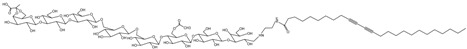	[18]
	Ba^2+^	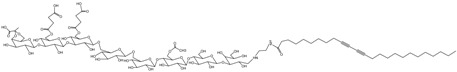	[19]
β-cyclodextrin	arginine, lysine	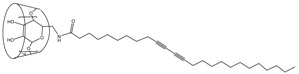	[20]
G_M1_ gaglioiside	cholera toxin	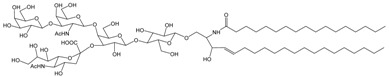	[21,22,27]
G_Tb1_ gaglioiside	botulinum neurotoxin	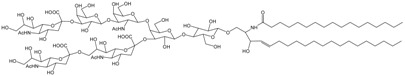	[21]
β-glucoside	*E. coli*	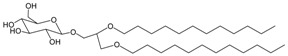	[23]
	*E. coli*	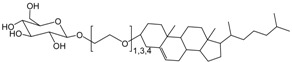	[24]
*N*-acetamide-β-glucoside	lectin	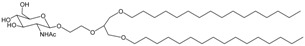	[25]
*β*-maltotriose	*E. coli*	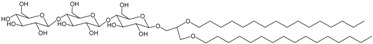	[26]
Sialic acid-*β*-glucoside	hemagglutinin (HA1)	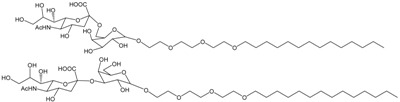	[28]

**Table 2:** Biomimetic Lipids-PDA systems for detection.

Biomimetic PDA	Detection Fields	Ref
DMPC ^1^/PDA	Phospholipase action	[32,33,34,35]
	Penetration enhancers (azone, oleic acid etc.)	[39]
	Casein oligomers	[40]
	α-Lactoalbumin, β-lactoglobulin	[41]
DMPE ^2^, DMPC/PDA	Antimicrobial peptides (mellitin, maganin, alamethicin, polymixin B)	[36,37,45]
DMPC,sphingomyelin,cholesterol/PDA	Polymixin B	[45]
	Bacterial fingerprinting	[46]
DMPC,DMPE,DGS ^3^,DMPG ^4^/PDA	Lipoproteins	[44]
	Plasma molecular components	[47]
DMPC, DMPE, DMPG/PDA giant vesicles	*Vaccina* virus, polymyxin B, lipophilic pharmaceutical (nortriptyline)	[48]
PIP_2_^5^/PDA	Aminoglycoside antibiotics (neomycin)	[38]
Sphingomyelin/PDA	Sphingomyelinase action	[34]
Cerebroside/PDA	Galactosidase action	[34]
Cholesterol/PDA	SLO ^6^	[43]

^1^ dimyristoyl phosphatidylcholine; ^2^ dimyristoyl phosphoethanolamine; ^3^ 1,2-dipalmitoyl-sn-glycero-3-succinate; ^4^ 1,2-dimyristoyl-sn-glycero-3-phosphorylglycerol; ^5^ phosphatidylinositol-4,5-phospholipids; ^6^ streptolysine O.

## References

[1] Okada S., Peng S., Spevak W., Charych D. (1998). Color and chromism of polydiacetylene vesicles. Acc. Chem. Res..

[2] UkáYe B., MináBaik J., HwaáKim M. (2014). Unprecedented colorimetric responses of polydiacetylenes driven by plasma induced polymerization and their patterning applications. Chem. Commun..

[3] Lu S., Jia C., Duan X., Zhang X., Luo F., Han Y., Huang H. (2014). Polydiacetylene vesicles for hydrogen peroxide detection. Colloids Surf. A.

[4] Sarkar A., Okada S., Matsuzawa H., Matsuda H., Nakanishi H. (2000). Novel polydiacetylenes for optical materials: Beyond the conventional polydiacetylenes. J. Mater. Chem..

[5] Ahn D.J., Kim J.-M. (2008). Fluorogenic polydiacetylene supramolecules: Immobilization, micropatterning, and application to label-free chemosensors. Acc. Chem. Res..

[6] Lee S., Kim J.-Y., Chen X., Yoon J. (2016). Recent progress in stimuli-induced polydiacetylenes for sensing temperature, chemical and biological targets. Chem. Commun..

[7] Stencel-Baerenwald J.E., Reiss K., Reiter D.M., Stehle T., Dermody T.S. (2014). The sweet spot: Defining virus-sialic acid interactions. Nat. Rev. Microbiol..

[8] Kovbasnjuk O. (2005). New insights into the role of Shiga toxins in intestinal disease. Gastroenterology.

[9] Nobile-Orazio E., Carpo M., Scarlato G. (1994). Gangliosides. Drugs.

[10] Goñi F.M. (2014). The basic structure and dynamics of cell membranes: An update of the Singer–Nicolson model. Biochim. Biophys. Acta.

[11] Charych D.H., Nagy J.O., Spevak W., Bednarskit M.D. (1993). Direct colorimetric detection of a receptor-ligand interaction by a polymerized bilayer assembly. Science.

[12] Charych D.H., Spevak W., Nagy J.O., Bednarski M.D. (1992). Specific Interaction of Influenza Virus with Organized Assemblies of Polydiacetylenes. MRS Online Proc. Libr. Arch..

[13] Reichert A., Nagy J.O., Spevak W., Charych D. (1995). Polydiacetylene liposomes functionalized with sialic acid bind and colorimetrically detect influenza virus. J. Am. Chem. Soc..

[14] Lio A., Reichert A., Ahn D.J., Nagy J.O., Salmeron M., Charych D.H. (1997). Molecular imaging of thermochromic carbohydrate-modified polydiacetylene thin films. Langmuir.

[15] Ma B., Fan Y., Zhang L., Kong X., Li Y., Li J. (2003). Direct colorimetric study on the interaction of *Escherichia coli* with mannose in polydiacetylene Langmuir–Blodgett films. Colloids Surf. B.

[16] Zhang Y., Fan Y., Sun C., Shen D., Li Y., Li J. (2005). Functionalized polydiacetylene-glycolipid vesicles interacted with *Escherichia coli* under the TiO_2_ colloid. Colloids Surf. B.

[17] Leal M.P., Assali M., Fernández I., Khiar N. (2011). Copper-Catalyzed Azide–Alkyne Cycloaddition in the Synthesis of Polydiacetylene: “Click Glycoliposome” as Biosensors for the Specific Detection of Lectins. Chemistry.

[18] Yun D., Jeong D., Cho E., Jung S. (2015). Colorimetric detection of some highly hydrophobic flavonoids using polydiacetylene liposomes containing pentacosa-10, 12-diynoyl succinoglycan monomers. PLoS ONE.

[19] Yun D., Cho E., Dindulkar S.D., Jung S. (2016). Succinoglycan Octasaccharide Conjugated Polydiacetylene-Doped Alginate Beads for Barium (II) Detection. Macromol. Mater. Eng..

[20] Cho E., Kim H., Choi Y., Paik S.R., Jung S. (2016). Polydiacetylenyl β-cyclodextrin based smart vesicles for colorimetric assay of arginine and lysine. Sci. Rep..

[21] Charych D., Cheng Q., Reichert A., Kuziemko G., Stroh M., Nagy J.O., Spevak W., Stevens R.C. (1996). A ‘litmus test’ for molecular recognition using artificial membranes. Chem. Biol..

[22] Pan J.J., Charych D. (1997). Molecular recognition and colorimetric detection of cholera toxin by poly (diacetylene) liposomes incorporating G_m1_ ganglioside. Langmuir.

[23] Ma Z., Li J., Liu M., Cao J., Zou Z., Tu J., Jiang L. (1998). Colorimetric detection of *Escherichia coli* by polydiacetylene vesicles functionalized with glycolipid. J. Am. Chem. Soc..

[24] Ma Z., Li J., Jiang L., Cao J., Boullanger P. (2000). Influence of the Spacer Length of Glycolipid Receptors in Polydiacetylene Vesicles on the Colorimetric Detection of *Escherichia coli*. Langmuir.

[25] Guo C.X., Boullanger P., Liu T., Jiang L. (2005). Size effect of polydiacetylene vesicles functionalized with glycolipids on their colorimetric detection ability. J. Phys. Chem. B.

[26] Su Y.L., Li J.R., Jiang L., Cao J. (2005). Biosensor signal amplification of vesicles functionalized with glycolipid for colorimetric detection of *Escherichia coli*. J. Colloid Interface Sci..

[27] Cheng Q., Stevens R.C. (1997). Monolayer properties of monosialioganglioside in the mixed diacetylene lipid films on the air/water interface. Chem. Phys. Lipids.

[28] Deng J., Sheng Z., Zhou K., Duan M., Yu C.-Y., Jiang L. (2009). Construction of effective receptor for recognition of avian influenza H5N1 protein HA1 by assembly of monohead glycolipids on polydiacetylene vesicle surface. Bioconjugate Chem..

[29] Van den Heuvel M., Löwik D.W., van Hest J.C. (2010). Effect of the diacetylene position on the chromatic properties of polydiacetylenes from self-assembled peptide amphiphiles. Biomacromolecules.

[30] Schott M. (2006). The colors of polydiacetylenes: A commentary. J. Phys. Chem. B.

[31] Yoon B., Lee S., Kim J.-M. (2009). Recent conceptual and technological advances in polydiacetylene-based supramolecular chemosensors. Chem. Soc. Rev..

[32] Jelinek R., Okada S., Norvez S., Charych D. (1998). Interfacial catalysis by phospholipases at conjugated lipid vesicles: Colorimetric detection and NMR spectroscopy. Chem. Biol..

[33] Okada S.Y., Jelinek R., Charych D. (1999). Induced color change of conjugated polymeric vesicles by interfacial catalysis of phospholipase A_2_. Angew. Chem. Int. Ed..

[34] Rozner S., Kolusheva S., Cohen Z., Dowhan W., Eichler J., Jelinek R. (2003). Detection and analysis of membrane interactions by a biomimetic colorimetric lipid/polydiacetylene assay. Anal. Biochem..

[35] Nie Q., Zhang Y., Zhang J., Zhang M. (2006). Immobilization of polydiacetylene onto silica microbeads for colorimetric detection. J. Mater. Chem..

[36] Kolusheva S., Shahal T., Jelinek R. (2000). Peptide—Membrane interactions studied by a new phospholipid/polydiacetylene colorimetric vesicle assay. Biochemistry.

[37] Kolusheva S., Boyer L., Jelinek R. (2000). A colorimetric assay for rapid screening of antimicrobial peptides. Nat. Biotechnol..

[38] Jung H.-S., Ahn N., Lee J., Seo S., Suh K.-Y., Kim J., Kim K. (2012). Biomimetic detection of aminoglycosidic antibiotics using polydiacetylene–phospholipids supramolecules. Chem. Commun..

[39] Evrard D., Touitou E., Kolusheva S., Fishov Y., Jelinek R. (2001). A new colorimetric assay for studying and rapid screening of membrane penetration enhancers. Pharm. Res..

[40] Sokolovski M., Sheynis T., Kolusheva S., Jelinek R. (2008). Membrane interactions and lipid binding of casein oligomers and early aggregates. Biochim. Biophys. Acta.

[41] De Oliveira C.P., Soares N.D.F.F., Fontes E.A.F., de Oliveira T.V., Maradini Filho A.M. (2012). Behaviour of polydiacetylene vesicles under different conditions of temperature, pH and chemical components of milk. Food Chem..

[42] Kolusheva S., Wachtel E., Jelinek R. (2003). Biomimetic lipid/polymer colorimetric membranes molecular and cooperative properties. J. Lipid Res..

[43] Ma G., Cheng Q. (2005). Vesicular polydiacetylene sensor for colorimetric signaling of bacterial pore-forming toxin. Langmuir.

[44] Hanin-Avraham N., Fuhrman B., Mech-Dorosz A., Kolusheva S., Porgador A., Aviram M., Jelinek R. (2009). Lipoprotein interactions with chromatic membranes as a novel marker for oxidative stress-related diseases. Biochim. Biophys. Acta.

[45] Volinsky R., Kliger M., Sheynis T., Kolusheva S., Jelinek R. (2007). Glass-supported lipid/polydiacetylene films for colour sensing of membrane-active compounds. Biosens. Bioelectron..

[46] Scindia Y., Silbert L., Volinsky R., Kolusheva S., Jelinek R. (2007). Colorimetric detection and fingerprinting of bacteria by glass-supported lipid/polydiacetylene films. Langmuir.

[47] Kolusheva S., Yossef R., Kugel A., Katz M., Volinsky R., Welt M., Hadad U., Drory V., Kliger M., Rubin E. (2012). Array-based disease diagnostics using lipid/polydiacetylene vesicles encapsulated in a sol–gel matrix. Anal. Chem..

[48] Pevzner A., Kolusheva S., Orynbayeva Z., Jelinek R. (2008). Giant chromatic lipid/polydiacetylene vesicles for detection and visualization of membrane interactions. Adv. Funct. Mater..

[49] Peng H.-P., Lee K.H., Jian J.-W., Yang A.-S. (2014). Origins of specificity and affinity in antibody–protein interactions. Proc. Natl. Acad. Sci. USA.

[50] Su Y.-L., Li J.-R., Jiang L. (2004). Chromatic immunoassay based on polydiacetylene vesicles. Colloids Surf. B.

[51] Jiang L., Luo J., Dong W., Wang C., Jin W., Xia Y., Wang H., Ding H., Jiang L., He H. (2015). Development and evaluation of a polydiacetylene based biosensor for the detection of H5 influenza virus. J. Virol. Methods.

[52] Jeong J.-P., Cho E., Yun D., Kim T., Lee I.-S., Jung S. (2017). Label-Free Colorimetric Detection of Influenza Antigen Based on an Antibody-Polydiacetylene Conjugate and Its Coated Polyvinylidene Difluoride Membrane. Polymers.

[53] Park C.H., Kim J.P., Lee S.W., Jeon N.L., Yoo P.J., Sim S.J. (2009). A Direct, Multiplex Biosensor Platform for Pathogen Detection Based on Cross-linked Polydiacetylene (PDA) Supramolecules. Adv. Funct. Mater..

[54] Kwon I.K., Kim J.P., Sim S.J. (2010). Enhancement of sensitivity using hybrid stimulus for the diagnosis of prostate cancer based on polydiacetylene (PDA) supramolecules. Biosens. Bioelectron..

[55] Won S.H., Sim S.J. (2012). Signal enhancement of a micro-arrayed polydiacetylene (PDA) biosensor using gold nanoparticles. Analyst.

[56] Lim M.-C., Shin Y.-J., Jeon T.-J., Kim H.-Y., Kim Y.-R. (2011). Microbead-assisted PDA sensor for the detection of genetically modified organisms. Anal. Bioanal. Chem..

[57] Jung S.-H., Jang H., Lim M.-C., Kim J.-H., Shin K.-S., Kim S.M., Kim H.-Y., Kim Y.-R., Jeon T.-J. (2015). Chromatic biosensor for detection of phosphinothricin acetyltransferase by use of polydiacetylene vesicles encapsulated within automatically generated immunohydrogel beads. Anal. Chem..

[58] Xia Y., Deng J., Jiang L. (2010). Simple and highly sensitive detection of hepatotoxin microcystin-LR via colorimetric variation based on polydiacetylene vesicles. Sens. Actuators B.

[59] De Oliveira T.V., de FF Soares N., Coimbra J.S.D.R., de Andrade N.J., Moura L.G., Medeiros E.A., de Medeiros H.S. (2015). Stability and sensitivity of polydiacetylene vesicles to detect Salmonella. Sens. Actuators B.

[60] Gill I., Ballesteros A. (2003). Immunoglobulin–Polydiacetylene Sol–Gel Nanocomposites as Solid-State Chromatic Biosensors. Angew. Chem..

[61] Lee S.W., Kang C.D., Yang D.H., Lee J.S., Kim J.M., Ahn D.J., Sim S.J. (2007). The development of a generic bioanalytical matrix using polydiacetylenes. Adv. Funct. Mater..

[62] Pindzola B.A., Nguyen A.T., Reppy M.A. (2006). Antibody-functionalized polydiacetylene coatings on nanoporous membranes for microorganism detection. Chem. Commun..

[63] Kim K.-W., Choi H., Lee G.S., Ahn D.J., Oh M.-K. (2008). Effect of phospholipid insertion on arrayed polydiacetylene biosensors. Colloids Surf. B.

[64] Kolusheva S., Kafri R., Katz M., Jelinek R. (2001). Rapid Colorimetric Detection of Antibody—Epitope Recognition at a Biomimetic Membrane Interface. J. Am. Chem. Soc..

[65] Kang D.H., Jung H.-S., Lee J., Seo S., Kim J., Kim K., Suh K.-Y. (2012). Design of polydiacetylene-phospholipid supramolecules for enhanced stability and sensitivity. Langmuir.

[66] Seo S., Lee J., Choi E.J., Kim E.J., Song J.Y., Kim J. (2013). Polydiacetylene Liposome Microarray Toward Influenza A Virus Detection: Effect of Target Size on Turn-On Signaling. Macromol. Rapid Commun..

[67] Matsubara T., Onishi A., Saito T., Shimada A., Inoue H., Taki T., Nagata K., Okahata Y., Sato T. (2010). Sialic acid-mimic peptides as hemagglutinin inhibitors for anti-influenza therapy. J. Med. Chem..

[68] Song S., Ha K., Guk K., Hwang S.-G., Choi J.M., Kang T., Bae P., Jung J., Lim E.-K. (2016). Colorimetric detection of influenza A (H1N1) virus by a peptide-functionalized polydiacetylene (PEP-PDA) nanosensor. RSC Adv..

[69] Cheng Q., Stevens R.C. (1997). Coupling of an induced fit enzyme to polydiacetylene thin films: Colorimetric detection of glucose. Adv. Mater..

[70] Kolusheva S., Shahal T., Jelinek R. (2000). Cation-Selective Color Sensors Composed of Ionophore−Phospholipid−Polydiacetylene Mixed Vesicles. J. Am. Chem. Soc..

[71] Jaworski J., Yokoyama K., Zueger C., Chung W.-J., Lee S.-W., Majumdar A. (2011). Polydiacetylene incorporated with peptide receptors for the detection of trinitrotoluene explosives. Langmuir.

[72] Jaworski J.W., Raorane D., Huh J.H., Majumdar A., Lee S.-W. (2008). Evolutionary screening of biomimetic coatings for selective detection of explosives. Langmuir.

[73] Kim T.H., Lee B.Y., Jaworski J., Yokoyama K., Chung W.-J., Wang E., Hong S., Majumdar A., Lee S.-W. (2011). Selective and sensitive TNT sensors using biomimetic polydiacetylene-coated CNT-FETs. ACS Nano.

[74] Wu J., Zawistowski A., Ehrmann M., Yi T., Schmuck C. (2011). Peptide functionalized polydiacetylene liposomes act as a fluorescent turn-on sensor for bacterial lipopolysaccharide. J. Am. Chem. Soc..

[75] Jiang H., Hu X.Y., Schlesiger S., Li M., Zellermann E., Knauer S.K., Schmuck C. (2017). Morphology-Dependent Cell Imaging by Using a Self-Assembled Diacetylene Peptide Amphiphile. Angew. Chem..

[76] Rangin M., Basu A. (2004). Lipopolysaccharide identification with functionalized polydiacetylene liposome sensors. J. Am. Chem. Soc..

[77] Weis W.I., Drickamer K. (1996). Structural basis of lectin-carbohydrate recognition. Annu. Rev. Biochem..

[78] Li Y., Wang L., Wen Y., Ding B., Sun G., Ke T., Chen J., Yu J. (2015). Constitution of a visual detection system for lead (II) on polydiacetylene–glycine embedded nanofibrous membranes. J. Mater. Chem. A.

[79] Ripoll M., Neuberg P., Kichler A., Tounsi N., Wagner A., Remy J.-S. (2016). PH-responsive nanometric polydiacetylenic micelles allow for efficient intracellular siRNA delivery. ACS Appl. Mater. Interfaces.

[80] Wang C., Ma Z. (2005). Colorimetric detection of oligonucleotides using a polydiacetylene vesicle sensor. Anal. Bioanal. Chem..

[81] Park M.-K., Kim K.-W., Ahn D.J., Oh M.-K. (2012). Label-free detection of bacterial RNA using polydiacetylene-based biochip. Biosens. Bioelectron..

[82] Walmsley J.A., Burnett J.F. (1999). A New Model for the K^+^-Induced Macromolecular Structure of Guanosine 5′-Monophosphate in Solution. Biochemistry.

[83] Lee J., Kim H.-J., Kim J. (2008). Polydiacetylene liposome arrays for selective potassium detection. J. Am. Chem. Soc..

[84] Lee J., Jun H., Kim J. (2009). Polydiacetylene–liposome microarrays for selective and sensitive mercury (II) detection. Adv. Mater..

[85] Jung Y.K., Kim T.W., Park H.G., Soh H.T. (2010). Specific Colorimetric Detection of Proteins Using Bidentate Aptamer-Conjugated Polydiacetylene (PDA) Liposomes. Adv. Funct. Mater..

[86] Wu W., Zhang J., Zheng M., Zhong Y., Yang J., Zhao Y., Wu W., Ye W., Wen J., Wang Q. (2012). An aptamer-based biosensor for colorimetric detection of *Escherichia coli* O157:H7. PLoS ONE.

[87] Wen J.T., Bohorquez K., Tsutsui H. (2016). Polydiacetylene-coated polyvinylidene fluoride strip aptasensor for colorimetric detection of zinc (II). Sens. Actuators B.

[88] Bayer E.A., Wilchek M. (1990). [14] Protein biotinylation. Methods Enzymol..

[89] Jung Y.K., Park H.G., Kim J.-M. (2006). Polydiacetylene (PDA)-based colorimetric detection of biotin–streptavidin interactions. Biosens. Bioelectron..

[90] Jung Y.K., Kim T.W., Jung C., Cho D.Y., Park H.G. (2008). A polydiacetylene microchip based on a biotin–streptavidin interaction for the diagnosis of pathogen infections. Small.

[91] Li L., An X., Yan X. (2015). Folate-polydiacetylene-liposome for tumor targeted drug delivery and fluorescent tracing. Colloids Surf. B.

[92] Weitman S.D., Lark R.H., Coney L.R., Fort D.W., Frasca V., Zurawski V.R., Kamen B.A. (1992). Distribution of the folate receptor GP38 in normal and malignant cell lines and tissues. Cancer Res..

[93] Wang D.-E., Wang Y., Tian C., Zhang L., Han X., Tu Q., Yuan M., Chen S., Wang J. (2015). Polydiacetylene liposome-encapsulated alginate hydrogel beads for Pb^2+^ detection with enhanced sensitivity. J. Mater. Chem. A.

[94] Ma X., Sheng Z., Jiang L. (2014). Sensitive naked-eye detection of Hg^2+^ based on the aggregation and filtration of thymine functionalized vesicles caused by selective interaction between thymine and Hg^2+^. Analyst.

